# Whole Exome Sequencing to Identify Genetic Variants Associated with Raised Atherosclerotic Lesions in Young Persons

**DOI:** 10.1038/s41598-017-04433-x

**Published:** 2017-06-22

**Authors:** James E. Hixson, Goo Jun, Lawrence C. Shimmin, Yizhi Wang, Guoqiang Yu, Chunhong Mao, Andrew S. Warren, Timothy D. Howard, Richard S. Vander Heide, Jennifer Van Eyk, Yue Wang, David M. Herrington

**Affiliations:** 1 Human Genetics Center, UTHealth School of Public Health, Houston, TX 77030 USA; 2 Department of Electrical and Computer Engineering, Virginia Polytechnic Institute and State University, Arlington, VA 22203 USA; 3 Biocomplexity Institute of Virginia Tech, Virginia Tech, Blacksburg, VA 24061 USA; 4 Center for Genomics & Personalized Medicine Research, Wake Forest School of Medicine, Winston-Salem, NC 27157 USA; 5 Department of Pathology, Louisiana State University Health Science Center, New Orleans, LA 70112 USA; 6 Advanced Clinical BioSystems Research Institute, Heart Institute and Department of Medicine, Cedars-Sinai Medical Center, Los Angeles, CA 90048 USA; 7 Department of Internal Medicine, Wake Forest School of Medicine, Winston-Salem, NC 27157 USA

## Abstract

We investigated the influence of genetic variants on atherosclerosis using whole exome sequencing in cases and controls from the autopsy study “Pathobiological Determinants of Atherosclerosis in Youth (PDAY)”. We identified a PDAY case group with the highest total amounts of raised lesions (n = 359) for comparisons with a control group with no detectable raised lesions (n = 626). In addition to the standard exome capture, we included genome-wide proximal promoter regions that contain sequences that regulate gene expression. Our statistical analyses included single variant analysis for common variants (MAF > 0.01) and rare variant analysis for low frequency and rare variants (MAF < 0.05). In addition, we investigated known CAD genes previously identified by meta-analysis of GWAS studies. We did not identify individual common variants that reached exome-wide significance using single variant analysis. In analysis limited to 60 CAD genes, we detected strong associations with COL4A2/COL4A1 that also previously showed associations with myocardial infarction and arterial stiffness, as well as coronary artery calcification. Likewise, rare variant analysis did not identify genes that reached exome-wide significance. Among the 60 CAD genes, the strongest association was with NBEAL1 that was also identified in gene-based analysis of whole exome sequencing for early onset myocardial infarction.

## Introduction

Coronary artery disease (CAD) due to atherosclerosis remains a major health burden across the globe. Atherosclerosis is a life-long process that involves accumulation of lipids, inflammatory cells, and smooth muscle cells in the intima of the arterial wall to form atherosclerotic lesions that can block blood circulation required for transport of oxygen and critical nutrients to the heart. Population-based epidemiological studies identified important risk factors for CAD such as elevated low density lipoprotein cholesterol (LDL-C) and reduced high density lipoprotein cholesterol (HDL-C). Genetic factors also influence CAD risk, but the identity of the responsible genes still remains unclear. Attempts to identify genes that influence CAD began with association studies of DNA variants in biological candidate genes from metabolic pathways with known involvement in atherosclerosis like cholesterol transport and metabolism^1^. More recently, genetic studies of CAD have relied on genome wide association studies (GWAS) that test millions of genetic variants (single nucleotide polymorphisms) across the genome for associations in large case-control studies^2^.

Despite these efforts, the identification of genes that influence CAD has remained elusive. A major reason is that clinical CAD is a heterogenous disease, resulting from many different pathophysiologic mechanisms. In addition, important subclinical measures of CAD like extent of atherosclerosis are difficult to measure in human populations. To address these problems, a multicenter autopsy study was established to provide direct measurements of atherosclerotic lesions called “Pathobiological Determinants of Youth (PDAY)”. PDAY obtained arterial measurements of subclinical atherosclerosis in young persons (15–34 years of age) who died of external causes unrelated to heart disease (e.g., accidents, homicide, suicide). Results of PDAY studies directly demonstrated the atherogenic effects of exposure to risk factors such as elevated plasma levels of cholesterol and LDL levels, as well as smoking and hypertension^3^.

In this study, we are employing PDAY to find genetic variants that are associated with a quantitative measure of subclinical CAD, the involvement of arterial surfaces with complicated raised lesions. We selected a case group with the highest amounts of arterial raised lesions for comparisons with a control group with no detectable raised lesions. Our goal was to identify genetic variants that are enriched in the case group (risk alleles) or the control group (protective alleles). To identify genetic variants, we are using whole exome sequencing by isolation of protein coding regions from individual genomes, followed by next generation sequencing. While GWAS examines associations of relatively common genetic variants, whole exome sequencing can detect low frequency and rare variants that are specific to the study population and may have larger effects on disease phenotypes. A novel aspect of this whole exome sequencing study is the inclusion of genome-wide proximal promoter regions that contain sequence elements that regulate gene expression^4^.

In addition to using whole exome sequencing for variant discovery in PDAY, we were also interested in relating our results for subclinical CAD with other population-based genetic studies of clinical CAD. In particular, we compared our results with whole exome sequencing for early onset myocardial infarction (NIH Exome Sequencing Project) to provide replication of their results, and to identify genes that are unique to each study^5^. Likewise, we investigated known genes that have previously shown strong and replicable associations with CAD from GWAS studies^2^. These CAD-associated genes represent numerous physiological and molecular processes, including well known pathways such as lipoprotein transport and metabolism, as well as pathways that have not been previously implicated in CAD risk.

## Results

### PDAY Subject Characteristics

Table 1 shows characteristics of 359 cases and 626 controls that were selected from the PDAY cohort to find genes harboring variations associated with atherosclerosis in young persons by whole exome sequencing. The cases had approximately 100-fold higher percentages of surface involvement in raised lesions across multiple arteries (total raised lesions) including thoracic and abdominal aorta, and coronary artery. Overall, African American and European American groups were similarly represented, but more males than females were included in the study reflecting the sampling history of the parent PDAY study.

**Table 1:** Characteristics of PDAY subjects (FS, fatty streaks; RL, raised lesions).

	European Americans				African Americans			
	Control		Case		Control		Case	
	Male	Female	Male	Female	Male	Female	Male	Female
Sample size	207	87	150	42	272	60	137	30
Age	24.52 ± 5.27	28.01 ± 4.20	26.35 ± 5.14	29.10 ± 3.88	27.36 ± 4.24	28.27 ± 4.00	26.95 ± 4.55	28.17 ± 4.22
Total Raised Lesions	0.27 ± 0.76	0.40 ± 0.92	24.52 ± 20.62	33.79 ± 23.27	0.38 ± 0.80	0.21 ± 0.63	30.60 ± 27.10	36.09 ± 34.61
LDL + VLDL	140.33 ± 50.43	133.49 ± 50.64	161.98 ± 61.77	166.17 ± 77.81	122.75 ± 50.85	122.10 ± 37.65	153.80 ± 65.77	151.24 ± 47.66
HDL	48.97 ± 17.89	54.83 ± 20.61	52.97 ± 21.37	56.90 ± 21.04	57.31 ± 23.54	63.62 ± 27.85	55.91 ± 24.13	52.53 ± 19.15
BMI	24.64 ± 4.00	24.41 ± 4.73	26.42 ± 5.81	24.45 ± 6.42	24.76 ± 4.21	25.23 ± 6.14	25.78 ± 6.08	24.71 ± 5.65
Thoracic Aorta FS	17.10 ± 12.35	16.14 ± 11.17	22.69 ± 13.40	24.10 ± 14.91	23.22 ± 14.29	22.67 ± 13.52	29.95 ± 16.08	21.95 ± 12.58
Thoracic Aorta RL	0.01 ± 0.13	0.00 ± 0.00	1.08 ± 3.11	1.19 ± 4.37	0.01 ± 0.12	0.05 ± 0.41	2.01 ± 5.06	4.99 ± 13.34
Abdominal Aorta FS	22.67 ± 17.29	34.38 ± 21.62	29.05 ± 17.89	37.62 ± 16.02	27.42 ± 20.46	36.84 ± 21.35	34.57 ± 20.99	35.58 ± 18.22
Abdominal Aorta RL	0.09 ± 0.45	0.29 ± 0.86	11.81 ± 13.03	22.14 ± 17.05	0.18 ± 0.59	0.08 ± 0.30	14.98 ± 16.19	24.71 ± 18.27
Coronary Artery FS	2.03 ± 3.82	2.73 ± 6.63	7.02 ± 8.50	8.47 ± 11.42	5.25 ± 9.88	3.20 ± 5.02	11.99 ± 15.31	12.98 ± 15.38
Coronary Artery RL	0.16 ± 0.59	0.11 ± 0.38	11.64 ± 16.92	10.46 ± 18.45	0.18 ± 0.50	0.08 ± 0.35	13.61 ± 18.84	6.40 ± 16.49

### Whole exome sequencing Results

**Figure 1:**
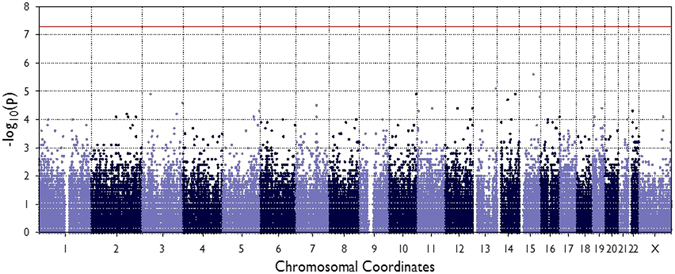
Manhattan plot for single variant analysis. The Y-axis shows −log_10_(p) values for common variants (MAF > 0.01) and the X-axis shows chromosomal positions for each variant. The threshold for statistical significance after correction for multiple testing (4.3 × 10^−7^) is shown by the red horizontal line.

We used whole exome sequencing to identify genetic variants in coding regions of the genome that are associated with atherosclerotic raised lesions using a case/control study design. We also added proximal promoter regions across the genome that contain regulatory sequence elements^4^. Association tests for single variants were used for relatively common variants with minor allele frequencies greater than 0.01 (MAF > 0.01). These association tests used linear mixed-model regression with covariates including age, gender, race, sequencing platform, and the top three principal components. Figure 1 shows a Manhattan plot from the single-variant association tests, where the X-axis shows genetic variant positions across the chromosomes and the Y-axis shows the negative log of p-values so that higher values represent stronger significance levels. The horizontal red line shows the threshold value for exome-wide significance (p < 4.3 × 10^−7^). Supplementary Figure 1 shows the quantile-quantile (Q-Q) plot for the single variant tests, demonstrating a good fit of the observed to expected significance values after applying the covariates. A well-calibrated Q-Q plot follows the diagonal line, while an inflated (off-diagonal) plot suggests either possible batch effect or population effects. These results shows that these effects were successfully controlled in our statistical analyses. Table 2 shows the 20 genes and variants that yielded the strongest associations, although none reached exome-wide significance (p < 4.4 × 10^−6^). We also selected single variant association results for the variants in proximal promoter regions only (Q-Q plot in Supplementary Figure 2). We observed modest enrichments compared to the all variant results, suggesting possible functional contributions from promoter variants, but did not find any associations that met exome-wide significance.

**Table 2:** Top hits for single variant analysis (MAF > 0.01).

Chr	Position	SNP	MAF	P-value	Beta	rsID	Gene
15	66188031	C/G	0.015	2.61E-06	0.421	rs16949083	MEGF11,RAB11A
13	111102865	G/A	0.097	7.36E-06	0.169	rs72657934	COL4A2
3	42799858	T/A	0.498	1.20E-05	−0.0830	rs339678	CCDC13,HIGD1A
10	134137638	G/A	0.022	1.32E-05	0.298	rs11146329	STK32C
14	88453424	G/A	0.066	1.35E-05	0.170	rs2119703	GALC
15	100649248	G/A	0.067	1.61E-05	−0.174	rs61752832	ADAMTS17
14	52741950	A/G	0.426	1.78E-05	0.0961	rs810633	PTGDR
3	197300254	A/G	0.201	2.76E-05	−0.106	rs268611	BDH1
7	98889954	T/A	0.017	3.48E-05	0.356	rs116972017	MYH16
11	72287350	A/G	0.294	3.90E-05	−0.0905	rs1299161	PDE2A
12	60173356	A/T	0.222	4.09E-05	−0.104	rs3763980	SLC16A7
19	44455262	G/C	0.082	4.24E-05	0.157	rs73038529	ZNF221
19	44610843	C/T	0.066	4.31E-05	0.176	rs72480796	ZNF224
12	132325239	G/A	0.337	4.42E-05	−0.0915	rs6598163	MMP17
5	176083175	T/G	0.478	4.87E-05	−0.0788	rs6878977	TSPAN17
Y	10036453	T/G	0.134	4.96E-05	0.166	rs79596466	RNA5-8SP6
22	23256260	T/G	0.123	5.01E-05	0.134	rs455941	IGLC4,IGLC5,IGLJ4,IGLJ5,IGLJ6
11	5566620	A/G	0.013	5.19E-05	0.393	rs56291963	HBG2,OR52H1
3	169813340	C/T	0.404	6.35E-05	0.0839	rs7643249	PHC3
2	173451074	T/C	0.011	7.02E-05	0.402	rs36014095	PDK1

For low-frequency and rare variants with MAF < 0.05, we performed gene-based tests to assess contributions of rare variants within the same gene. In gene-based tests, variants residing in the same gene are grouped together to assess whether they are collectively associated with a phenotype. We used two commonly used methods for gene-based tests: a collapsing method (CMC)^6^ and SKAT^7^. Supplementary Figure 3 shows Q-Q plots for each method using low frequency and rare variants with MAF < 0.05. Table 3 shows the 10 genes with the strongest associations using CMC and SKAT, but none reached exome-wide significance (p < 2.2 × 10^−6^).

**Table 3:** Top 10 genes from gene-based analysis (CMC and SKAT) of low frequency and rare variants (MAF < 0.05).

Rank	CMC				SKAT			
	Gene	Chr	Region	P-value	Gene	Chr	Region	P-value
1	VAX2	2	71127544–71160222	4.10E-05	VAX2	2	71127544–71160222	6.42E-05
2	ZBTB10	8	81398083–81431555	8.05E-05	ZBTB10	8	81398083–81431555	0.00013045
3	ULK2	17	19683921–19746538	8.30E-05	PLLP	16	57292435–57318739	0.00018174
4	SCAF4	21	33043815–33074643	0.0001171	PTPRA	20	2903792–3016333	0.00032945
5	ZBTB16	11	113931024–114117994	0.0004213	LEKR1	3	156543960–156763438	0.00042348
6	USP9X	X	41025425–41075744	0.0004422	CDH11	16	64981824–65155772	0.0004925
7	ZNF883	9	115774593–115774637	0.0004631	DUSP5P1	1	228785805–228785811	0.00053582
8	MICALL2	7	1474260–1498873	0.000471	SCAF4	21	33043815–33074643	0.00078692
9	ARMC8	3	137906007–138003375	0.0004865	TSKS	19	50243197–50266714	0.00080308
10	PPEF2	4	76782011–76823716	0.0005027	ZNF883	9	115774593–115774637	0.00098536

### Results for CAD Genes Selected from GWAS

**Figure 2:**
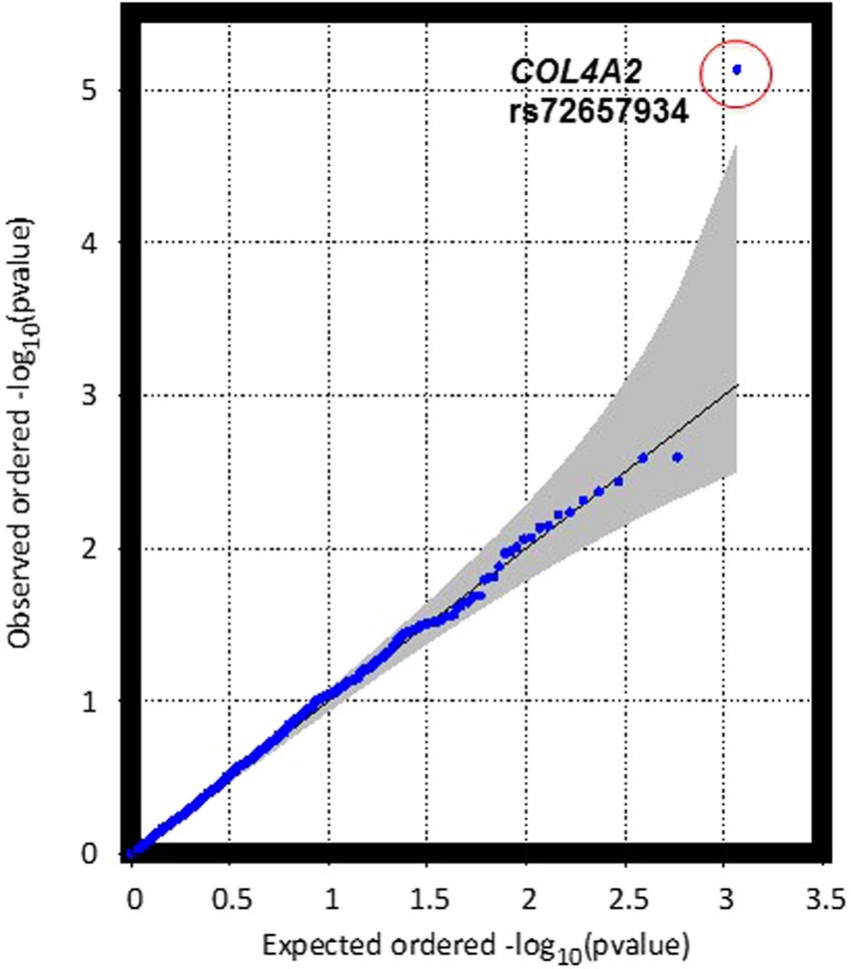
Q-Q plot for single variant analysis of CAD GWAS genes. This Q-Q plot shows observed versus expected ordered −log_10_(p) values for single variant analysis of CAD GWAS genes (MAF > 0.01), with the shaded region showing standard errors.

**Figure 3:**
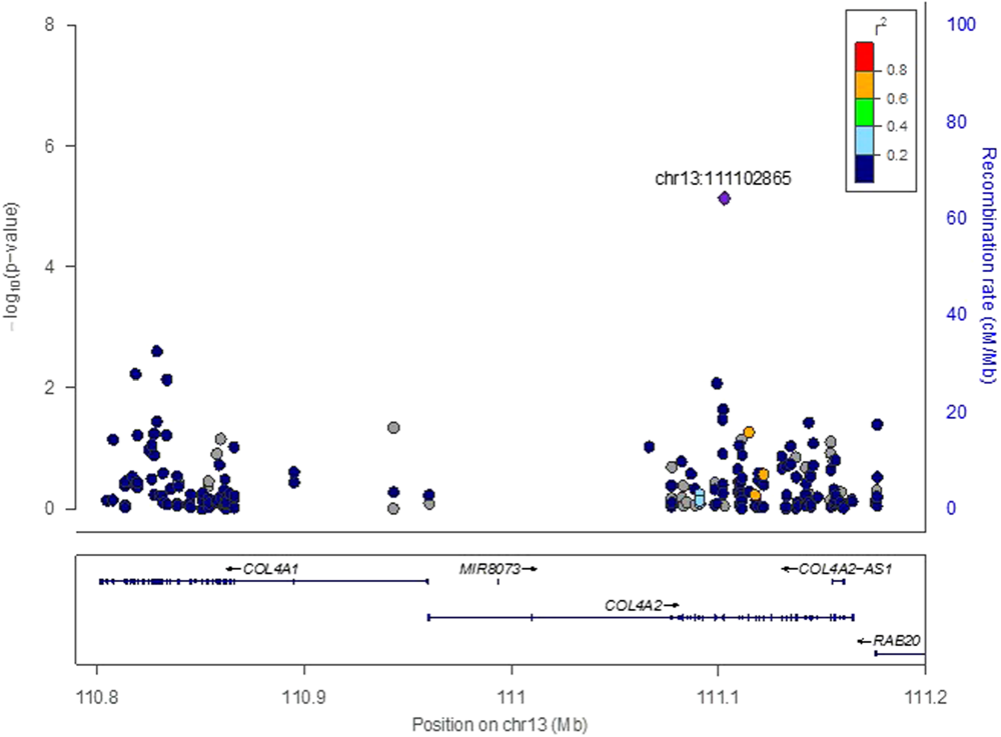
Regional Zoom Plot for chromosomal 13 region containing COL4A1 and COL4A2. This Regional Zoom Plot shows the positions (X-axis) and −log_10_(p) values (Y-axis) for variants in COL4A1 and COL4A2 (maps below plot), LD among variants (colors of dots), and recombination rates (blue curve) for this region of chromosome 13.

To investigate whether genes that have previously shown consistent associations with coronary artery disease (CAD) are associated with atherosclerosis in young persons, we selected 60 genes from published meta-analysis of GWAS of CAD^2^. Figure 2 shows a Q-Q plot of 1,166 single variant tests (MAF > 0.01) from these 60 genes only. We identified one significant association with raised lesions for a variant (rs72657934) in the gene encoding alpha-2 subunit of type IV collagen (COL4A2) after correcting for multiple tests (p = 7.4 × 10^−6^ that reached the Bonferroni significance threshold of 4.3 × 10^−5^). Table 4 shows the 10 genes and variants that showed the strongest associations from the 60 known CAD genes. Figure 3 presents a regional plot from the whole exome sequencing results showing patterns of linkage disequilibrium in the region of chromosome 13 containing COL4A1 and COL4A2. The X-axis shows the position of variants across this chromosomal region, and the Y-axis shows the negative log of p-values for association with each variant. The color of the dots show the linkage disequilibrium values (r^2^) with the index variant (rs72657934). We performed conditional analyses on selected variants to test for independence of the association with rs72657934 in COL4A2. Conditioning on rs3742207 (Gln1134His) or rs1562173 (our top hit in COL4A1) did not weaken p-values for rs72657934 (p = 4.7 × 10^−6^ and p = 4.2 × 10^−6^, respectively), indicating that association with our top significant variant (rs72657934) is independent of previously identified GWAS variants. We also used gene-based analysis for low frequency and rare variants (MAF < 0.05) in the 60 CAD GWAS genes. Table 5 shows the top results for CMC and SKAT gene-based analyses in 60 CAD genes.

**Table 4:** Top ten hits for single variant analysis of CAD GWAS gene variants.

Chr	Position	SNP	MAF	P-value	Beta	rsID	Gene
13	111102865	G/A	0.09719	7.36E-06	0.169	rs72657934	COL4A2
13	110828922	G/A	0.27868	0.002531	−0.0714	rs1562173	COL4A1
15	91434277	C/T	0.04818	0.002561	0.1526	rs2229074	FES
10	90984990	G/A	0.11574	0.003676	0.0955	rs2297472	LIPA
10	91007360	T/G	0.19645	0.004237	0.0750	rs1051338	LIPA
19	45409167	C/G	0.22698	0.004882	−0.0612	rs440446	APOE,TOMM40
13	29041007	A/T	0.04162	0.005791	0.146	rs3751398	FLT1
13	110818598	T/G	0.26294	0.006018	−0.0640	rs3742207	COL4A1
1	57111169	C/G	0.27381	0.00716	0.0252	rs61772962	PPAP2B,PRKAA2
13	110833702	C/T	0.23081	0.007345	−0.0717	rs16975492	COL4A1

**Table 5:** Top genes from gene-based analysis (CMC and SKAT) of low frequency and rare variants (MAF < 0.05) for CAD GWAS genes.

Rank	CMC				SKAT			
	Chr	Region	Gene	P-value	Chr	Region	Gene	P-value
1	2	203879503–204078281	NBEAL1	0.002461	1	109859485–109940735	SORT1	0.017836
2	17	17398825–17399882	RASD1	0.003532	6	12716955–13273088	PHACTR1	0.021258
3	1	55504976–55529215	PCSK9	0.0244	17	1968415–2203563	SMG6	0.041314
4	17	1968415–2203563	SMG6	0.0496	17	17398825–17399882	RASD1	0.058087
5	11	116660857–116663319	APOA5	0.06693	11	116660857–116663319	APOA5	0.10293
6	2	44065936–44104993	ABCG8	0.07264	6	160906980–160906980	LPAL2	0.11367
7	10	44793346–44876259	CXCL12	0.08058	2	85811391–85820239	VAMP5	0.1166
8	17	17409125–17495090	PEMT	0.09814	5	131705219–131729935	SLC22A5	0.13411
9	2	203760853–203776965	WDR12	0.1038	1	55504976–55529215	PCSK9	0.18973
10	12	111856493–111886068	SH2B3	0.1154	15	91411730–91425038	FURIN	0.19594

## Discussion

Previous studies have identified genes containing variants that are associated with clinical CAD, yet the major portion of CAD genetic heritability remains to be explained^2^. In this study, we are using PDAY for genetic studies to reduce the phenotypic heterogeneity inherent in clinical CAD, and to provide quantitative measures of subclinical CAD (raised atherosclerotic lesions) that accumulate over time and can eventually result in clinical CAD. We performed whole exome sequencing in 626 controls and 359 cases with raised lesions as directly measured by pathologists in arterial specimens in the PDAY study (Table 1). The whole exome sequencing strategy was to sequence PDAY cases and controls for coding regions in the genome to identify DNA variants that could alter functional properties of proteins (amino acid substitutions). We also isolated proximal promoter regions to identify DNA variants that could alter regulation of gene expression across the genome.

After sequencing, we used single variant statistical analysis for relatively common genetic variants (minor allele frequency >0.01) across the exome to identify associations with arterial raised lesions. However, we did not identify any variants that achieved exome-wide significance after adjustments for multiple testing (Fig. 1, Table 2). This result may reflect a truly polygenic basis of inheritance for atherosclerosis, with contributions from large numbers of genes each with only small additive effects. This polygenic basis may mean that only very large studies would have sufficient statistical power to achieve exome-wide significance after correction for multiple testing in association tests. We also performed gene-based analyses (CMC and SKAT) for low frequency and rare variants with MAF < 0.05, but did not identify genes that reached the threshold for exome-wide significance after correction for multiple testing (Table 3). This result indicates that rare variants may not exert strong genetic effects on atherosclerosis, a finding that is now being reported by large scale whole exome and whole genome sequencing projects for other complex diseases liked type 2 diabetes^8^.

In addition to standard whole exome sequencing, we used custom probes to capture genome-wide proximal promoter regions (200 bp upstream). However, single variant analysis of promoter regions did not find strong evidence for associations (Supplementary Figure 2). An exception is VAX2 that showed associations in gene-based analysis (CMC and SKAT) only with inclusion of a rare variant in the promoter region (Table 3). VAX2 on chromosome 2 encodes Ventral Anterior Homeobox 2, a transcription factor involved in eye and forebrain development that is also expressed in cardiac and arterial tissues^9, 10^. The VAX2 transcription factor is a dominant/negative regulator of expression of Wnt signaling antagonists like TCF7L2^10^. Therefore, VAX2 would likely act to regulate gene expression in atherogenesis via pathways like Wnt signaling, rather than as a structural protein in lesion development. Interestingly, TCF7L2 has been associated with Type 2 diabetes in several GWAS, and was subsequently found to be associated with CAD severity in diabetic and non-diabetic subjects^11^. Wnt signaling is involved in heart valve development, regulating genes from osteogenesis pathways that are also important in coronary artery calcification^12^. Perhaps VAX2 is also involved in development of other anatomic structures like the arterial wall. Our results suggest that the significant gene-based association for VAX2 relied on inclusion of rs557150817 (C/A) in the upstream promoter region. The rare A variant was found in 8 cases and one control. This variant may alter VAX2 expression via disruption of transcription factor binding sites located in this region (Egr1 and SP1), potentially altering expression of downstream targets of VAX2 transcription factor activities.

We also performed separate analyses of CAD genes identified in GWAS that tested millions of variants across the genome with much larger numbers of subjects than our current study^2^. These genes have shown consistent and replicable associations with CAD in GWAS of many different cohorts. We found that variant rs72657934 in COL4A2 (intron 20) was prominent in common variant analysis as shown in the Q-Q plot (Fig. 2, Table 4), exceeding the significance threshold for this separate analysis (p < 4 × 10^−5^, after Bonferroni correction for 1,667 variants). This COL4A2 variant also showed the second lowest p-value in exome-wide single variant analysis (Table 2). Interestingly, we found associated variants in COL4A1, including a missense variant (rs3742007) that causes Gln to His substitution at amino acid position 1134 (Table 4). This missense variant has shown associations with myocardial infarction in Japanese subjects^13^, and arterial stiffness in Sardinian subjects^14^. Associations of COL4A1/COL4A2 reported from meta-analysis of GWAS for coronary artery calcification are of special interest since these genes also showed associations with myocardial infarct in contrast with most other loci associated with coronary artery calcification^15^. Our top hit among CAD GWAS genes (rs72657934) appears to act independently in single variant tests, since conditional analysis with other variants in COL4A1/COL4A2 did not decrease the significance levels for rs72657934 associations. Furthermore, linkage disequilibrium does not appear to be responsible for rs72657934 associations, since linkage disequilibrium values are low among the COL41/COL4A2 variants as shown in the regional plot (Fig. 3).

The COL4A1/COL4A2 genes are situated head-to-head on chromosome 13, and share the same promoter elements (Fig. 3). These genes encode type IV collagen alpha subunits that interact to form triple helices that are major constituents of basement membranes in many tissues, including vascular tissues. Potential functional mechanisms for effects of COL4A1/COL4A2 variation have been demonstrated in a recent study of rs3742207 in intron 3 (A/G) that showed association with CAD in GWAS but that was not captured in our whole exome sequencing^2^. Primary cultures of vascular smooth muscle cells and endothelial cells showed the G allele was associated with lower expression levels of both COL4A1 and COL4A2 due to lower transcriptional activity^16^. In addition, primary smooth cells from GG homozygotes had higher rates of apoptosis and lower amounts of collagen IV, as well as thinner fibrous caps that are typically found in plaques prone to sudden rupture. In the current study, the minor rs72657934 A allele was associated with raised lesions (beta = 0.169, Table 4). Although the potential function of this variant is not known, its location in a noncoding region (intron 20) suggests effects on gene expression rather than alteration of protein structure. The previous functional study of rs3742207 suggests that amounts or proportions of type 4 collagen chains can affect pathophysiological attributes of atherosclerotic lesions. It should be noted that type IV collagen also has non-structural properties potentially involved in atherosclerosis. *In vitro* studies have shown that canstatin, the non-collagenous 1 domain of the alpha-2 chain, can inhibit proliferation of endothelial cells and induce apoptosis^17^.

In addition, we performed separate analyses to investigate rare variants that may underlie associations of CAD genes but that were not included in GWAS with common variants. The results differed between CMC and SKAT analyses, with lower p-values emerging from CMC. Our top gene from CMC was NBEAL1, a gene that showed associations in the previous NHLBI Exome Sequencing Project^5, 18^. In meta-analysis of GWAS, WDR12 located adjacent to NBEAL1 showed significant associations with CAD. A subsequent study showed WDR12 contains a major e-QTL for NBEAL1 expression in aortic media^19^. Our gene-based analysis (CMC) of WDR12 also showed associations with raised lesions, although p-values did not reach statistical significance. NBEAL1 is comprised of 25 exons spanning chromosome 2q, and encodes the neurobeachin-like 1 protein that was first isolated from a brain cDNA library^20^. NBEAL1 is highly expressed in glioma, with potential involvement in membrane-processing signals. NBEAL1 also is expressed in heart and artery, but little is known about any functional role in these tissues. The distribution of rare variants did not offer any substantive clues concerning their functional effects, since potentially deleterious variants were found among both cases and controls.

While the PDAY study provides direct measurements of atherosclerosis in arterial specimens, the unique nature of these measures hinders direct comparisons of our results with other cohorts. Therefore, we have compared our results with genetic studies of clinical sequelae of atherosclerosis like myocardial infarct and early onset myocardial infarction, and other subclinical measures such as coronary artery calcification. Interestingly, of the 60 CAD genes identified by meta-analysis of GWAS, we only found significant association with COL4A1/COL4A2 encoding alpha-1 and alpha-2 subunits of type IV collagen. Perhaps the effects of COL4A1/COL4A2 variation occur early in development of atherosclerosis, setting the stage for effects of other genetic variants in subsequent phases of atherogenesis.

In summary, we used whole exome sequencing of PDAY cases with raised atherosclerotic lesions and controls, but did not find novel associations with exome-wide significance for either common variants with MAF > 0.01 or low frequency and rare variants with MAF < 0.05. This result may be due to a truly polygenic basis of inheritance for atherosclerosis, requiring very large studies to achieve exome-wide significance for multiple variants with only small additive effects. Addition of proximal promoter regions to coding sequences did not substantially alter our findings, except for VAX2 that showed associations in gene-based analysis only with inclusion of the promoter region. In separate analyses of 60 CAD genes identified by meta-analysis of GWAS, we confirmed previously reported associations only with common variants in COL4A1/COL4A2 on chromosome 13. Likewise, in rare variant analysis of CAD genes, we found associations with NBEAL1 on chromosome 2 that was initially detected by GWAS associations with WDR12 that contains an e-QTL for NBEAL1 expression in aortic media, and that was independently identified in gene-based analyses from the NIH Exome Sequencing Project for early onset myocardial infarction^5, 18^. These data emphasize the potential causal contributions of genetic variation in NBEAL1 and COL4A1/COL4A2 to the pathogenesis of premature atherosclerosis.

## Methods

### Study Subjects

Cases and controls were selected from autopsied PDAY subjects (15–34 years of age) who died of non-CAD related causes such as accident, homicide, and suicide. The subjects included male and female European Americans and African Americans (Table 1). The 359 cases were selected according to the highest total raised lesion scores determined by the sum of percentages of surface involvement in raised lesions from thoracic and abdominal aorta, and coronary arteries as previously described^21^. The 626 controls were selected from PDAY subjects with no arterial raised lesions with frequency matching for age, race, and gender. All methods and experimental studies were carried out in accordance with relevant guidelines and regulations after approval by the Committee for the Protection of Human Subjects (IRB) at the University of Texas Health Science Center at Houston. The PDAY subjects were not consented since the post-mortem samples were collected at autopsy by County Coroners’ Offices after death due to external causes (homicide, suicide, accident)^21^.

### Measures of Arterial Atherosclerosis

Arterial trees from PDAY subjects were collected at autopsy and treated with Sudan IV that stains lipids on the arterial surface. The percentage of involvement of arterial surfaces with fatty streaks and raised lesions was estimated by averaging independent values from three pathologists as previously described^21^.

### Whole exome sequencing

Genomic DNA samples were purified from archived PDAY liver tissues using an automated paramagnetic bead based extraction system (Promega Magnasil). The genomic DNA samples were used for exome capture with TargetSeq reagents (Life Technologies, Inc.) based on high density oligonucleotide hybridization of GENCODE annotated coding exons, NCBI CCDS, exon flanking sequences (including intron splice sites), small non-coding RNAs (e.g., microRNAs) and a selection of miRNA binding sites. We also designed custom probes to capture promoter regions located within ~200 bp upstream from transcriptional start sites that typically contain the majority of transcription factor binding sites^4^. These custom capture probes included proximal promoter regions for 18,661 genes after excluding pseudogenes, unidentified LOC genes, repetitive non-coding RNAs (tRNAs, ribosomal RNAs), and intractable repeat regions (polynucleotides and Alu repeats). After capture, we used automated library construction (AB Library Builder, Life Technologies, Inc.) with addition of barcodes for multiplexed sequencing. We began sequencing with the oligonucleotide ligation-based SOLiD 5500xl platform (Life Technologies, Inc.) that performs massively parallel sequencing of individual DNA molecules amplified on beads affixed to glass slides (288 subjects). For the remaining 697 subjects, we used the Ion Proton platform (Life Technologies, Inc.), based on proton assays for polymerase sequencing of individual DNA molecules in wells of modified semiconductor chips. For both platforms, we sequenced each case with their corresponding controls in the same sequencing run (SOLiD slide, Proton chip). We constructed BAM files (CUSHAW for SOLiD, tmap1 aligner for Proton) for variant calling using Freebayes algorithms, and annotated the resulting VCF files using VEP. We created a single VCF by merging the calls from each sequencing platform, and filled in the reference sequences (non-variable positions) using a jointly called VCF from the two platforms.

### Statistical Analysis

For all association analyses, we used linear mixed-model analyses from the EPACTS software pipeline (http://genome.sph.umich.edu/wiki/EPACTS) to adjust for population stratification and to minimize batch effects from sequencing platforms by calculating the kinship matrix between samples from the exome VCF file. EPACTS supports linear mixed-model based tests for both single variant tests (common variants) and gene-based tests (low frequency and rare variants). Age, gender, and race were used as covariates in all statistical tests. First, we evaluated association signals of individual common variants with minimum minor allele frequencies (MAF) of 0.01 and with minimum minor allele count of 5. We used the ‘q.emmax’ test in the EPACTS pipeline, which is an implementation of the linear mixed-model^22^. We treated case/control status as a quantitative trait after adjusting for covariates. We then tested associations of low frequency and rare variants (MAF < 0.05) using mixed-model gene-based tests to find whether functional rare variants in each gene are jointly associated with the phenotype. Variants were annotated by Variant Effect Predictor (VEP)^23^ with gene symbols and functional effects, and only missense, nonsense, and splice variants with MAF less than 0.05 were included in the gene-based tests. We used two different EMMAX-based rare variant tests: the collapsing test (emmaxCMC) and the kernel-based test (mixed model SKAT) to test different hypotheses. The collapsing test assumes all functional rare variants in the gene would have effects of the same direction^6^, while the kernel-based test assumes that each variant can have either positive or negative effect to the phenotype^7^. Single-variant and gene-based test results were then sub-selected using the list of candidate genes from CAD GWAS^2^. For single-variant results, we also ran conditional analysis to evaluate dependence of our top hit from the candidate region (rs4773144) to known GWAS variants in COL4A1/COL4A2. All statistical tests used Bonferroni corrections to account for multiple testing.

## Electronic supplementary material

Supplemental Materials
