# Supplementary material for: Whole Exome Sequencing to Identify Genetic Variants Associated with Raised Atherosclerotic Lesions in Young Persons
Source: Sci Rep. 2017 Jun 22;7:4091. doi: 10.1038/s41598-017-04433-x (PMC5481334; doi:10.1038/s41598-017-04433-x)

SUPPLEMENTAL MATERIAL

Whole Exome Sequencing to Identify Genetic Variants Associated with Raised Atherosclerotic Lesions in Young Persons

James Hixson, Goo Jun, Lawrence Shimmin, Yizhi Wang, Guoqiang Yu, Chunhong Mao, Andrew Warren, Timothy Howard, Richard Vander Heide, Jennifer Van Eyk, Yue (Joseph) Wang, and David Herrington

SUPPLEMENTAL FIGURES AND LEGENDS

Supplementary Figure 1. **Q-Q plot for single variant analysis.** This Q-Q plot shows observed versus expected ordered $-\log_{10}(p)$ values for single variant analysis (MAF > 0.01), with the shaded region showing standard errors.

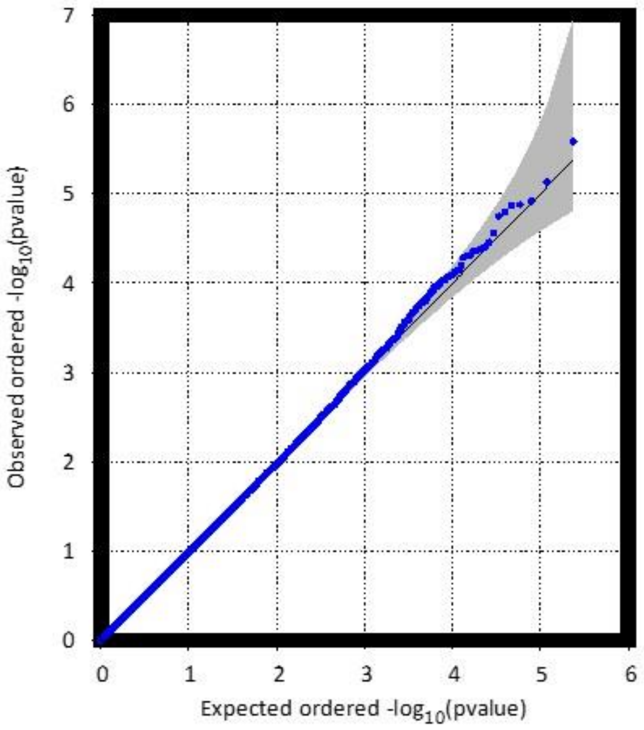

Supplementary Figure 2. Q-Q plot for single variant analysis of promoter regions only. This Q-Q plot shows observed versus expected ordered $-\log_{10}(p)$ values for single variant analysis (MAF > 0.01) of promoter regions only, with the shaded region showing standard errors.

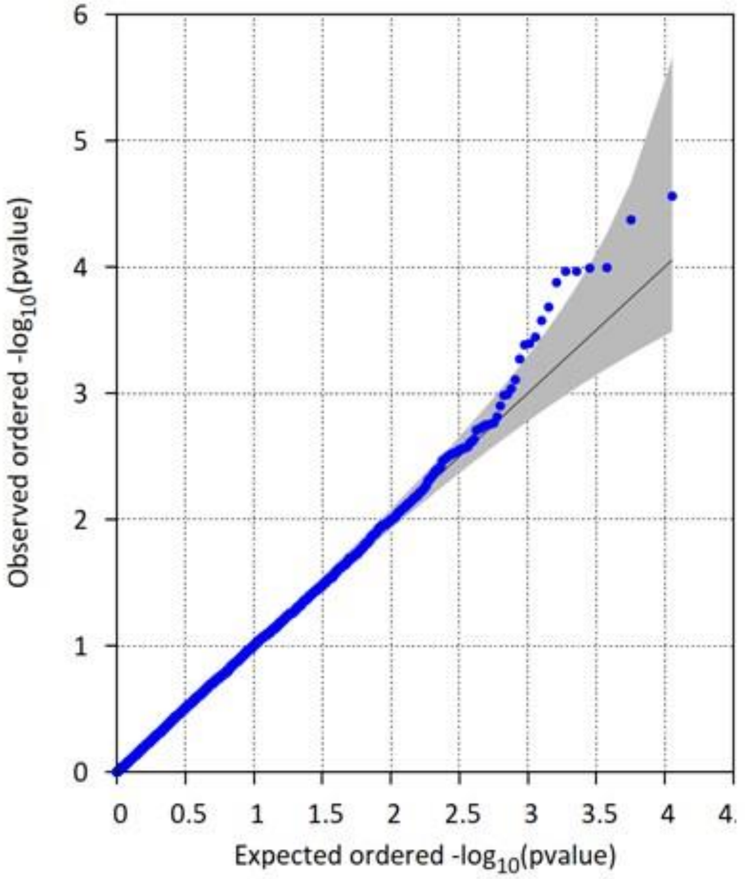

Supplementary Figure 3. Q-Q plots for rare variant analysis. Panel A shows the Q-Q plot for observed versus expected ordered $-\log_{10}(p)$ values for CMC analysis of low frequency and rare variants (MAF < 0.05), with the shaded region showing standard errors. Panel B shows the Q-Q plot for SKAT analysis (MAF< 0.05).

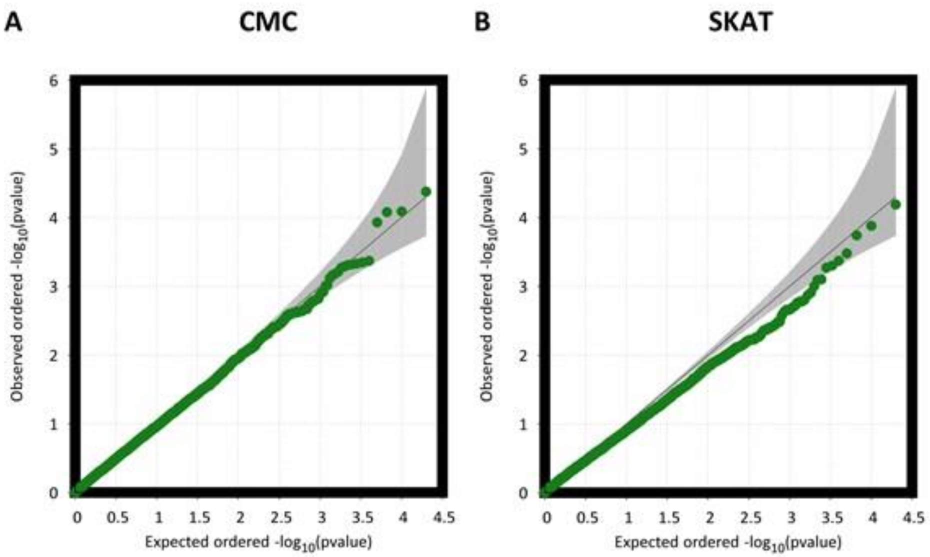

Supplement: Supplementary file 1 — Supplemental Materials [file 41598_2017_4433_MOESM1_ESM.pdf]
